# Retrospective 8-Year Study on the Antibiotic Resistance of Uropathogens in Children Hospitalised for Urinary Tract Infection in the Emilia-Romagna Region, Italy

**DOI:** 10.3390/antibiotics10101207

**Published:** 2021-10-04

**Authors:** Susanna Esposito, Giuseppe Maglietta, Margherita Di Costanzo, Martina Ceccoli, Gianluca Vergine, Claudio La Scola, Cristina Malaventura, Alice Falcioni, Alessandra Iacono, Antonella Crisafi, Lorenzo Iughetti, Maria Luisa Conte, Luca Pierantoni, Claudia Gatti, Caterina Caminiti, Giacomo Biasucci, on behalf of the UTI-Ped-ER Study Group

**Affiliations:** 1 Pediatric Clinic, University Hospital, Department of Medicine and Surgery, University of Parma, 43126 Parma, Italy; 2 Research and Innovation Unit, University Hospital, 43126 Parma, Italy; gmaglietta@ao.pr.it (G.M.); ccaminiti@ao.pr.it (C.C.); 3 Paediatrics and Neonatology Unit, Guglielmo da Saliceto Hospital, 29122 Piacenza, Italy; m.dicostanzo@ausl.pc.it (M.D.C.); g.biasucci@ausl.pc.it (G.B.); 4 Paediatrics Unit, Department of Medical and Surgical Sciences of Mothers, Children and Adults, University of Modena and Reggio Emilia, 41125 Modena, Italy; martina.ceccoli90@gmail.com (M.C.); lorenzo.iughetti@unimore.it (L.I.); 5 Paediatrics Unit, Rimini Hospital, AUSL Romagna, 47921 Rimini, Italy; gianluca.vergine@auslromagna.it (G.V.); marialuisa.conte@auslromagna.it (M.L.C.); 6 Paediatric Clinic, IRCCS Azienda Ospedaliero-Universitaria di Bologna, 40138 Bologna, Italy; clasc1976@gmail.com; 7 Paediatric Clinic, University of Ferrara, 44124 Ferrara, Italy; mlvcst@unife.it; 8 Paediatric Unit, Forlì Hospital, AUSL Romagna, 47121 Forlì, Italy; alice.falcioni@auslromagna.it; 9 Paediatrics and Neonatology Unit, Ravenna Hospital, AUSL Romagna, 48121 Ravenna, Italy; alessandra.iacono@auslromagna.it; 10 Paediatrics Unit, Santa Maria Nuova Hospital, AUSL-IRCCS of Reggio Emilia, 42123 Reggio Emilia, Italy; antonella.crisafi@ausl.re.it; 11 Paediatric Emergency Unit, IRCCS Azienda Ospedaliero-Universitaria di Bologna, 40138 Bologna, Italy; luca.pierantoni@aosp.bo.it; 12 Paediatric Surgery, University Hospital, 43126 Parma, Italy; clgatti@ao.pr.it

**Keywords:** antibiotic therapy, antimicrobial resistance, extended-spectrum beta-lactamase-producing bacteria, extensively drug-resistant bacteria, multidrug resistance, urinary tract infection

## Abstract

The development and spread of antibiotic resistance is an increasingly important global public health problem, even in paediatric urinary tract infection (UTI). In light of the variability in the data, it is necessary to conduct surveillance studies to determine the prevalence of antibiotic resistance in specific geographical areas to optimize therapeutic management. In this observational, retrospective, multicentre study, the medical records of 1801 paediatric patients who were hospitalised for UTI between 1 January 2012, and 30 June 2020, in Emilia-Romagna, Italy, were analysed. *Escherichia coli* was the most frequently detected pathogen (75.6%), followed by *Klebsiella pneumoniae* (6.9%) and *Pseudomonas aeruginosa* (2.5%). Overall, 840 cases (46.7%) were due to antimicrobial-resistant uropathogens: 83 (4.7%) extended spectrum beta-lactamase (ESBL)-producing, 119 (6.7%) multidrug resistant (MDR) and 4 (0.2%) extensively drug resistant (XDR) bacteria. Empirical antibiotic therapy failed in 172 cases (9.6%). Having ESBL or MDR/XDR uropathogens, a history of recurrent UTI, antibiotic therapy in the preceding 30 days, and empirical treatment with amoxicillin or amoxicillin/clavulanate were significantly associated with treatment failure, whereas first-line therapy with third-generation cephalosporins was associated with protection against negative outcomes. In conclusion, the increase in the resistance of uropathogens to commonly used antibiotics requires continuous monitoring, and recommendations for antibiotic choice need updating. In our epidemiological context, amoxicillin/clavulanate no longer seems to be the appropriate first-line therapy for children hospitalised for UTI, whereas third-generation cephalosporins continue to be useful. To further limit the emergence of resistance, every effort to reduce and rationalise antibiotic consumption must be implemented.

## 1. Introduction

Urinary tract infections (UTIs) are among the most common infections in children. Indeed, it is estimated that 11.3% of females and 3.6% of males develop at least one episode of a UTI within the first 16 years of life [1]. The prevalence of UTIs has a bimodal trend, with a first peak within the first year of life and a second between two and four years of age, corresponding to toilet training [2]. Moreover, in the first 6 months of life, uncircumcised males have a 10–12 times greater risk of UTIs than females. However, after the first year of life, the relationship is reversed, with an increased risk for females that persists into adulthood [3]. Most paediatric UTIs are caused by Gram-negative microorganisms of the *Enterobacteriaceae* family, of which *Escherichia coli* is the most frequent, causing more than 70% of UTIs. Other frequent pathogens of the same family are *Klebsiella* spp., *Enterobacter* spp. and *Proteus* spp. Although *Pseudomonas aeruginosa* is a less frequent Gram-negative pathogen, it is often associated with more severe UTIs. Among rare cases of UTIs caused by Gram-positive microorganisms, those caused by *Enterococcus* spp. are most frequent [4,5].

The development and spread of antibiotic resistance are increasingly important global public health problems, even in UTI, with variable prevalence in different geographical areas [6]. In recent years, the incidence of uropathogen resistance to commonly used antibiotics for paediatric UTI has increased worldwide. In the USA, out of 368,398 isolates in children with UTIs between January 1999 and December 2011, 1.97% were identified as third-generation cephalosporin resistant, with an increase in all demographic and age groups [7]. In Turkey, comparing the data collected in a single paediatric institution from 2009 to 2014, it was shown that *E. coli* resistance during the study period increased for ampicillin from 47.1% to 89%, for trimethoprim-sulphamethoxazole from 44.8% to 56% and for nitrofurantoin from 5.3% to 15.1% [8]. The most frequent risk factors associated with the development of UTIs due to antimicrobial-resistant pathogens are the previous use of antibiotics and urinary tract abnormalities [7,8,9,10]. Nevertheless, data for the surveillance of antibiotic resistance in UTIs frequently do not distinguish between adult and paediatric populations, and in many cases, the data are limited to single resistance mechanisms of specific pathogens, predominantly extended-spectrum beta-lactamase (ESBL)-producing *E. coli*. Furthermore, the prevalence of ESBL-producing pathogens in paediatric UTIs varies from 0.5% to 50% across studies [9,10,11]. In light of the variability in the data in the literature, it is necessary to conduct surveillance studies to determine the prevalence of antibiotic resistance in paediatric UTIs in specific geographical areas to optimize therapeutic management. Accordingly, this study aimed to describe the prevalence of antibiotic resistance among uropathogens in paediatric patients with UTI who were hospitalised in the last 8 years in the paediatric units of the Emilia-Romagna Region, Italy, as well as risk factors and outcomes associated with the onset of UTI caused by resistant pathogens.

## 2. Methods

### 2.1. Study Design and Population

In this observational, retrospective, multicentre study, medical records for paediatric patients of both sexes and under the age of 18 years who were hospitalised for UTI between 1 January 2012, and 30 June 2020, in the paediatric wards of Emilia-Romagna, Italy, were analysed. The medical records were extrapolated on the basis of the following diagnosis-related group (DRG) codes and/or International Classification of Diseases 9th revision (ICD-9) diagnosis codes: DRG 322, infections of the kidney and urinary tract, age <18 years; ICD-9-CM 590.1, acute pyelonephritis; ICD-9-CM 590.3, cystic pyeloureteritis; ICD-9-CM 590.8, pyelonephritis, unspecified; ICD-9-CM 590.9: kidney infection, unspecified. Patients with congenital or acquired immunodeficiencies, undergoing immunosuppressive therapy, or with neoplastic diseases were excluded. Only patients with positive urine cultures as defined in the Italian Society of Paediatric Nephrology guidelines [11] and febrile UTI were included in the study. The study was approved by the Ethics Committee of the participating centres (AVEN Protocol number 904/2020/OSS/AOUPR), and the parents of the enrolled patients were contacted by phone by each centre to come and sign informed consent for study participation; patients aged >8 years signed informed assent.

### 2.2. Data Evaluation

All medical records of subjects under the age of 18 years who met the inclusion and exclusion criteria were retrospectively reviewed. The data collection was focused on demographic characteristics, remote and proximate pathological history, blood and urinary laboratory parameters at onset, radiological findings (i.e., kidney and urinary tract ultrasound, voiding cystoureterography, renal scintigraphy) and laboratory data following the acute episode, uroculture and antibiogram results, antibiotic therapies administered before admission, during hospitalisation and prescribed at discharge, trends in vital parameters and any procedures carried out.

Positive urocultures were defined as the identification of a single pathogenic species present at ≥10^5^ CFU/mL. These cases were then analyzed by measuring frequency and describing the susceptibility/resistance of the individual pathogens. Pathogens that hydrolysed penicillin and first- to third-generation cephalosporins and aztreonam were defined phenotypically as ESBL [12]. Criteria for the definitions of multidrug resistance (MDR) and extensive drug resistance (XDR) were identified according to the combined guidelines of the Clinical and Laboratory Standards Institute (CLSI), the European Centre for Disease Prevention and Control (ECDC) and the Centers for Disease Control and Prevention (CDC) [12]. MDR pathogens were defined as non-susceptible to one agent in three antimicrobial categories. Pathogens were considered XDR if non-susceptible to one agent in all but two categories.

Risk factors as well as clinical, laboratory and renal ultrasound findings associated with UTI due to susceptible and resistant pathogens were investigated. All antibiotic molecules administered were recorded, and the clinical cure rate for therapies was evaluated in the entire study population using the clinical improvement of the patient with the resolution of fever and symptoms as a definition of therapeutic success.

For data collection, a special form was used for each patient. In the case of patients with multiple accesses within the inclusion criteria, the last access was chosen.

### 2.3. Statistical Analysis

Statistical analysis was conducted using R software v. 4.0.1. Descriptive statistics, as absolute and relative frequencies, means and standard deviations, were used to summarize the data. Pearson χ^2^ and Fisher’s exact tests were used to assess differences on all categorical variables and compare the two periods of analysis (2012–2015 vs. 2016–2020). Considering the eight-year study periods, we compared the first four years of enrollment with the last four years of the study period. Independent samples *t*-tests and Wilcoxon–Mann–Whitney tests were employed to compare the distribution of continuous variables. For all tests, an alpha <0.05 was considered statistically significant.

Univariable logistic regression models were then used to assess the effect of select risk factors and the onset of UTIs caused by resistant pathogens, as well as failure of empirical first-line treatment. 

## 3. Results

Table 1 summarises the characteristics of the study population. Overall, 1801 children (mean age ± standard deviation [SD], 1.9 ± 3.5 years) were enrolled during the study period, of whom 627 (34.8%) were hospitalised in 2012–2015 and 1174 (65.2%) in 2016–2020. Among them, 920 (51.1%) were females, and 1297 (72.0%) were of Caucasian origin. The children hospitalised during the years 2016–2020 were significantly older, were more frequently Caucasian and less often had a history of recurrent UTI in comparison with those hospitalised during the years 2012–2015. 

Table 2 summarizes the most frequently detected pathogens according to the study period. *E. coli* (1361 cases, 75.6%) was the most frequently detected pathogen in both study periods, followed by *K. pneumoniae* (124 cases, 6.9%) and *P. aeruginosa* (45 cases, 2.5%). From 2012–2015 to 2016–2020, a significant increase in cases due to *E. coli* and a significant decrease in cases due to *P. aeruginosa* were observed.

Table 3 summarises antibiotic susceptibility according to years of hospitalisation. Overall, 840/1801 cases (46.7%) were due to antimicrobial-resistant uropathogens: 83 (4.7%) to ESBL, 119 (6.7%) to MDR and 4 (0.2%) to XDR bacteria. Amoxicillin resistance was observed in 840 (46.7%) cases, amoxicillin/clavulanate resistance in 610 (33.8%), trimethoprim-sulfametoxazole resistance in 391 (21.7%), third-generation cephalosporin resistance in 210 (11.8%), aminoglicosyde resistance in 66 (3.7%) and fluoroquinolone resistance in 4 (0.2%). Antimicrobial resistance increased significantly from 2012–2015 to 2016–2020 (41.9% vs. 49.1%; *p* < 0.001), though the prevalence of MDR uropathogens decreased. The most frequent ESBL pathogens were *E. coli* (62/83, 74.7%) and *K. pneumoniae* (10/83, 12.0%), and the most frequent MDR pathogens were *E. coli* (68/119, 57.1%), *P. aeruginosa* (12/119, 10.1%), *K. pneumoniae* (7/119, 5.9%) and *Proteus mirabilis* (6/119, 5.0%). XDR pathogens were *E. coli* (3/4) and *K. pneumoniae* (1/4).

Table 4 summarises the results of univariate analysis for the risk of being infected by resistant pathogens, ESBL or MDR/XDR uropathogens. A history of recurrent UTI, antibiotic prophylaxis and antibiotic therapy in the preceding 30 days were significantly associated with an increased risk of UTI due to antimicrobial-resistant, ESBL or XDR/MDR pathogens, whereas urological malformations were significantly associated with a risk of antimicrobial resistance other than ESBL, MDR and XDR as well as with XDR/MDR UTI.

No significant differences in clinical findings, blood exams or urinary exams between the groups were observed. The empirical therapy administered to the study population is provided in Table 5. Third-generation cephalosporins were the most frequently prescribed antibiotics during the whole study period, and a significant increase in the prescription of amoxicillin/clavulanate during the years 2016–2020 in comparison with the years 2012–2015 was observed.

Overall, empirical antibiotic therapy failed in 172 children (9.6%). Table 6 shows factors significantly associated with treatment failure. Having ESBL or MDR/XDR uropathogens, history of recurrent UTI, antibiotic therapy in the preceding 30 days, having pielectasis or nephromegaly at echography, and empirical treatment with amoxicillin or amoxicillin/clavulanate were significantly associated with treatment failure. Conversely, empirical first-line treatment with third-generation cephalosporins was associated with protection against a negative outcome. In the presence of ESBL and MDR/XDR strains, third-generation cephalosporins alone were always associated with treatment failure.

## 4. Discussion

This study shows that antimicrobial resistance among uropathogens is an increasing phenomenon in children hospitalised for UTI in Italy, with a remarkable prevalence of ESBL as well as MDR/XDR strains. In general, previous antibiotic administration, including for UTI prophylaxis, is associated with resistance to antibiotics. Interestingly, risk factors for empirical treatment failure included having ESBL or MDR/XDR uropathogens, history of recurrent UTI, antibiotic therapy in the preceding 30 days, and empirical treatment with amoxicillin or amoxicillin/clavulanate, whereas empirical treatment with third-generation cephalosporins was associated with protection against poor outcomes.

Awareness of upper UTI aetiology and variation in pathogen susceptibility to antibiotics is essential for the choice of an effective therapy. As previously reported, our study showed *E. coli* and *K. pneumoniae* to be the most frequent uropathogens in children hospitalised for UTI and those most commonly observed in cases involving resistance [13,14,15,16]. In our population, resistance to amoxicillin and amoxicillin clavulanate was high, whereas resistance to third-generation cephalosporins was detected in <10% of uropathogens, in line with findings described by other authors [16,17,18]. The prevalence of ESBL, MDR and XDR uropathogens (4.7%, 6.7% and 0.2%, respectively) was similar to that previously reported in Europe and the United States [18,19,20] and lower than that reported in Asian countries [21,22,23]. However, it is not possible to draw conclusions on the absence of increase in ESBL and XDR strains as well as in the decrease of MDR strains during the study period.

To assure the highest probability of bacterial eradication, official guidelines indicate which antibiotics must be used. Taking into account the increase in the incidence of ESBL cases, Italian guidelines were recently updated, and an amoxicillin/clavulanic acid combination was indicated as the drug of choice for paediatric UTI treatment, highlighting that first- to third-generation cephalosporins have no additional role in this regard, as they are, in many cases, ineffective against ESBL-producing strains [10]. This explains why our data showed a significant increase in the prescription of amoxicillin/clavulanate during the study period. In contrast, although updated in 2016 [24], the American Academic of Pediatrics guidelines did not change previous recommendations that included 1st- and third-generation cephalosporins as potentially effective for the treatment of paediatric first UTI [25]. Similar limitations seem to be present in the NICE guidelines that, despite being updated in 2018, still indicate cefalexin as a first-line drug and recommend the use of amoxicillin/clavulanic acid only when culture results are available and the pathogen is found to be susceptible to the combination [26]. Interestingly, in our study, empirical treatment with penicillins and beta-lactamase-resistant penicillins was associated with treatment failure, whereas the use of third-generation cephalosporins as empirical first-line therapy was associated with protection against poor outcomes, with important implications for clinical practice in Italy. These results suggest that the use of amoxicillin/clavulanate as first-line empirical therapy in children hospitalised for UTI should be carefully re-evaluated but that third-generation cephalosporins appear to remain a valid first-line therapeutic option. The use of aminoglycosides should be recommended in the case of ESBL pathogens, though carbapenems should be limited to MDR and XDR strains [27].

Proper identification of patients at increased risk of antibiotic resistance can reduce the risk of ineffective therapy. In our study population, the previous use of antibiotics was the most important risk factor associated with antibiotic resistance. Antibiotics are among the drugs most commonly prescribed to children in hospital and community settings [28,29]. Unfortunately, a great number of these antibiotic prescriptions are unnecessary or inappropriate, as shown by the evidence that these drugs are frequently given to children who do not have bacterial diseases or an infectious disease [30,31]. The high prevalence of amoxicillin-clavulanate resistance can be explained by the reported high prescription rate of this drug in children with common respiratory infections (i.e., pharyngotonsillitis and acute otitis media) in the Emilia-Romagna Region [32] and highlights the importance of antimicrobial stewardship projects for a more rational use of this drug [33,34]. Regarding UTIs, one of the most common causes of microbial selection and emergence of resistance is the administration of antibiotics for prophylaxis in children with recurrent UTI episodes, especially when a structural or functional abnormality of the urinary tract is diagnosed [35]. In most cases, prophylaxis is not proven to be beneficial for preventing new renal scarring in children and is no longer recommended in official guidelines [36]. Despite this lack of recommendation, it is still implemented in many cases and amoxicillin-clavulanate is the most frequently prescribed antibiotic for this purpose [10], promoting the emergence of resistance and the development of difficult-to-treat new UTI episodes [37].

Nevertheless, a number of studies suggest that resistant UTI can occur even in children without any risk factors [38]. Although the presence of resistant uropathogens is associated with poor outcomes and the risk of complications, in routine practice, the risk of serious immediate or long-term clinical problems due to discordant therapy is lower than expected [39,40]. Despite some exceptions, studies evaluating outcomes of paediatric UTIs according to the antibiotic therapy administered have shown that in a relevant number of cases, the outcome of children receiving drugs that are ineffective against the infectious pathogen in vitro did not differ from children given concordant therapy [41]. At least two factors might explain why febrile UTIs can be cured even in the absence of effective in vitro antibiotic therapy. A certain number of febrile UTIs can resolve spontaneously, as was demonstrated for other bacterial diseases [42]. Second, it cannot be excluded that the administered antibiotic can reach urine and renal parenchyma concentrations much higher than those in blood after usual doses and that are used to define resistance in vitro [43,44]. In both, it seems likely that, eradication of the pathogens at the site of infection can occur despite discordant therapy, at least in some cases.

Limitations of the study included its retrospective nature, the different number of patients evaluated in the two study periods and the fact that the research is limited to a definite epidemiologic context. However, the strict inclusion criteria and the long duration of the study period permit to draw important results on what happens in real life.

## 5. Conclusions

Despite being very common and well-studied, UTIs in paediatric patients remain challenging and this manuscript improves clinical knowledge on their management. We highlighted that the increase in resistance of uropathogens to commonly used antibiotics requires continuous monitoring of microbiological characteristics of UTIs and updating of recommendations for antibiotic choice. In our epidemiological context, amoxicillin/clavulanate no longer seems to be the appropriate first-line therapy for children hospitalised for UTI, whereas third-generation cephalosporins continue to be helpful. In order to selectively use cephalosporins so as to avoid increasing resistance to this class of antibiotics, in future studies using multivariate analysis it may be interesting to understand if a differentiated approach can be adopted using amoxicillin-clavulanate at the first UTI and cephalosporins in the presence of second and subsequent infections. The emergence of antibiotic resistance is an unavoidable phenomenon closely correlating with the use of antibiotics themselves. To limit the emergence of resistance, every effort to reduce and rationalise antibiotic consumption must be made. Increased use of antibiotic stewardship can be greatly effective in this regard.

## Figures and Tables

**Table 1:** Demographic characteristics of the children with urinary tract infections (UTIs) according to years of hospitalisation.

Characteristic	Hospitalisation Period 2012–2015 *n* = 627	Hospitalisation Period 2016–2020 *n* = 1174	*p* Value
Mean age (SD), years	1.7 (3.3)	2.1 (3.8)	0.04
Sex, *n.*			0.3
F	296 (47%)	585 (50%)	
M	331 (53%)	589 (50%)	
Ethnicity, *n*.			<0.001
Caucasian	395 (78%)	902 (83%)	
Other	11 (2.2%)	19 (1.7%)	
Prenatal pyelectasis, *n*.	83 (14%)	141 (12%)	0.3
Prematurity at birth, *n*.	54 (9.0%)	111 (9.7%)	0.6
Neonatal complications, *n*.	64 (11%)	122 (11%)	>0.9
Vescico-ureteral reflux, *n*.	58 (9.3%)	86 (7.3%)	0.2
Other urological malformations, *n*.	58 (9.8%)	95 (8.4%)	0.3
Urological surgery, *n*.	17 (2.8%)	49 (4.2%)	0.13
History of recurrent UTI ^1^, *n*.	69 (12%)	98 (8.6%)	0.029
Antibiotic therapy in the 30 days before enrolment, *n*.	72 (12%)	131 (11%)	0.7

^1^ Defined by at least 3 episodes in the preceding 6 months. SD: standard deviation.

**Table 2:** Aetiology of urinary tract infections according to years of hospitalisation.

Pathogen	Hospitalisation Period 2012–2015 *n* = 627	Hospitalisation Period 2016–2020 *n* = 1174	*p* Value
*Escherichia coli*, *n*.	453 (72.0%)	908 (77.0%)	0.017
*Proteus mirabilis*, *n*.	9 (1.4%)	18 (1.5%)	0.9
*Klebsiella pneumoniae, n*.	49 (7.8%)	75 (6.4%)	0.3
*Pseudomonas aeruginosa, n.*	22 (3.5%)	23 (2.0%)	0.045
*Enterococcus faecalis*, *n*.	14 (2.2%)	21 (1.8%)	0.5
*Enterobacter* spp., *n*.	7 (1.1%)	16 (1.4%)	0.7
*Citrobacter koseri,n*.	5 (0.8%)	8 (0.7%)	0.8
*Staphylococcus aureus,n*.	1 (0.2%)	4 (0.3%)	0.7
Group B *Streptococcus*, *n*.	2 (0.3%)	1 (<0.1%)	0.3
Other, *n*.	10 (1.7%)	13 (1.2%)	0.4
Missing, *n*.	55 (8.8%)	87 (7.4%)	0.6

**Table 3:** Antibiotic susceptibility among uropathogens isolated from children hospitalised for urinary tract infection according to years of hospitalisation.

Characteristic	Hospitalisation Period 2012–2015 *n* = 627	Hospitalisation Period 2016–2020 *n* = 1174	*p* Value
Antimicrobial susceptibility	364 (58.1%)	597 (50.9%)	<0.001
Antimicrobial resistance other than ESBL, MDR, XDR	180 (28.7%)	454 (38.7%)	<0.001
ESBL	26 (4.1%)	57 (4.9%)	0.7
MDR	56 (8.9%)	63 (5.4%)	0.001
XDR	1 (0.2%)	3 (0.3%)	>0.9

ESBL: extended-spectrum beta-lactamase; MDR: multidrug resistant; XDR: extensively drug resistant.

**Table 4:** Risk factors associated with antibiotic resistance among children hospitalised for urinary tract infection.

	Antimicrobial Resistance other than ESBL, MDR, XDR *n* = 634			ESBL *n* = 83			MDR/XDR *n* = 123		
Characteristic	OR	95% CI	*p* Value	OR	95% CI	*p* Value	OR	95% CI	*p* Value
Age	1.03	0.98, 1.08	0.18	1.02	0.96, 1.08	0.45	1.03	0.98, 1.08	0.18
Sex									
F				-	-		-	-	
M	1.16	0.80, 1.69	0.42	1.1	0.70, 1.71	0.68	1.16	0.80, 1.69	0.42
Recurrent UTI	4.31	2.70, 6.75	<0.001	2.57	1.40, 4.47	0.001	4.31	2.70, 6.75	<0.001
Antibiotic prophylaxis	5.34	2.74, 10.2	<0.001	2.65	1.18, 5.55	0.013	5.34	2.74, 10.2	<0.001
Antibiotic therapy in the preceding 30 days	2.83	1.76, 4.44	<0.001	2.08	1.13, 3.60	0.013	2.83	1.76, 4.44	<0.001
Vescico-ureteral reflux, *n*.	1.10	0.76, 1.63	0.63						
Other urological malformations	2.67	1.71, 4.09	<0.001	1.18	0.61, 2.10	0.6	2.67	1.71, 4.09	<0.001
Nephromegaly	1.14	0.39, 2.66	0.78	0.95	0.23, 2.64	0.93	1.14	0.39, 2.66	0.78
Pyelectasis	1.19	0.79, 1.77	0.41	0.93	0.57, 1.50	0.76	1.19	0.79, 1.77	0.41

CI, confidence interval; ESBL, extended-spectrum beta-lactamase; MDR, multidrug resistant; OR, odds ratio; XDR, extensively drug resistant.

**Table 5:** Empirical therapy administered to the study population.

Therapy	Hospitalisation Period 2012–2015 *n* = 627	Hospitalisation Period 2016–2020 *n* = 1174	*p* Value
Amoxicillin	14 (2.2%)	20 (1.7%)	0.4
Amoxicillin/clavulanate	130 (21%)	363 (31%)	<0.001
Ampicillin +.aminoglycosides	112 (18%)	185 (16%)	0.3
third-generation cephalosporins	284 (45%)	479 (41%)	0.066
third-generation cephalosporins + aminoglycosides	29 (4.6%)	60 (5.1%)	0.7
Aminoglycosides	12 (1.9%)	15 (1.3%)	0.3
Quinolones	1 (0.2%)	2 (0.2%)	>0.9
TMX-SMZ	2 (0.3%)	0 (0%)	0.12
Other therapies	17 (2.7%)	30 (2.6%)	0.8

TMX-SMZ: trimethoprim/sulfamethoxazole.

**Table 6:** Factors significantly associated with treatment failure.

Characteristic	OR	95% CI	*p* Value
Antimicrobial resistance other than ESBL, MDR/XDR	1.39	1.00, 1.93	0.051
ESBL pathogens	3.69	2.12–6.21	<0.001
MDR/XDR pathogens	4.07	2.57, 6.36	<0.001
Age	1.03	0.98, 1.07	0.21
Male sex	1.08	0.78, 1.48	0.65
Recurrent UTI	2.04	1.28, 3.16	0.002
Antibiotic prophylaxis	1.87	0.93, 3.56	0.064
Antibiotic therapy in the previous 30 days	1.75	1.12, 2.66	0.011
Vescico-ureteral reflux	1.17	0.79, 1.80	0.45
Other urological malformations	1.49	0.98, 2.22	0.055
Pielectasis	1.41	1.01, 1.96	0.045
Nephromegaly	2.85	1.01, 1.96	<0.001
Treatment with amoxicillin	5.06	2.38, 10.2	<0.001
Treatment with amoxicillin/clavulanate	1.67	1.20, 2.32	0.002
Treatment with ampicillin + aminoglycosides	0.99	0.64, 1.49	0.96
Treatment with third-generation cephalosporins	0.46	0.32, 0.65	<0.001
Treatment with third-generation cephalosporins + aminoglycosides	0.77	0.32, 1.57	0.51
Treatment with aminoglycosides	1.20	0.28, 3.50	0.77

CI: confidence interval; ESBL: extended-spectrum beta-lactamase MDR: multidrug resistant; OR, odds ratio; XDR: extensively drug resistant.

## Data Availability

The data presented in this study are available in this article.
